# Effects of Lipotoxicity in Brain Microvascular Endothelial Cells During Sirt3 Deficiency-Potential Role in Comorbid Alzheimer’s Disease

**DOI:** 10.3389/fnagi.2021.716616

**Published:** 2021-07-28

**Authors:** Alpna Tyagi, Carol Mirita, Iman Shah, P. Hemachandra Reddy, Subbiah Pugazhenthi

**Affiliations:** ^1^Rocky Mountain Regional VA Medical Center, Aurora, CO, United States; ^2^Department of Medicine, University of Colorado Anschutz Medical Campus, Aurora, CO, United States; ^3^Internal Medicine Department and Garrison Institute on Aging, Texas Tech University Health Sciences Center, Lubbock, TX, United States

**Keywords:** blood brain barrier, brain micro endothelial cells, lipotoxicity, inflammation, sirt3, microglia

## Abstract

Silence information regulator 3 (SIRT3) is an NAD^+^ dependent deacetylase enzyme that enhances the function of key mitochondrial proteins. We have earlier demonstrated that deletion of Sirt3 gene leads to downregulation of metabolic enzymes, mitochondrial dysfunction and neuroinflammation in the brain, the major causes of Alzheimer’s disease (AD). We also reported recently that Sirt3 gene deletion in Alzheimer’s transgenic mice leads to exacerbation of neuroinflammation, amyloid plaque deposition and microglial activation. AD often coexists with other brain lesions caused by comorbidities which can exert their deleterious effects through the neurovascular unit. This unit consists of brain microvascular endothelial cells (BMECs), end feet of astrocytes, and pericytes. BMECs are uniquely different from other vascular endothelial cells because they are glued together by tight-junction proteins. BMECs are in constant contact with circulating factors as they line the luminal side. Therefore, we hypothesized that vascular endothelial injury caused by comorbidities plays a significant role in neuroinflammation. Herein, we investigated the effects of lipotoxicity in BMECs and how Sirt3 deficiency facilitate the deleterious effects of lipotoxicity on them using *in vivo* and *in vitro* models. We observed decreases in the levels of SIRT3 and tight junction proteins in the brain samples of western diet-fed APP/PS1 mice. Similar observations were obtained with Alzheimer’s post-mortem samples. Exposure of BEND3 cells, mouse brain-derived Endothelial cells3, to a combination of high glucose and palmitic acid resulted in significant (*P* < 0.01-*P* < 0.001) decreases in the levels of SIRT3, claudin-5 and ZO-1. Induction of inflammatory mediators, including Cox-2, CXCL1, RANTES, and GADD45β was also observed in these treated cells. Interestingly, the induction was more with Sirt3-silenced BEND3 cells, suggesting that Sirt3 deficiency exacerbates inflammatory response. Palmitic acid was more potent in inducing the inflammatory mediators. Significant cytotoxicity and changes in microglial morphology were observed when cocultures of Sirt3-silenced BEND3 and Sirt3-silenced BV2 cells were exposed to palmitic acid. Transendothelial electrical resistance measurement with these cocultures suggested decreased barrier integrity. The findings of this study suggest that hyperlipidemia in comorbidities can compromise blood brain barrier integrity by inducing inflammatory mediators and decreasing tight junction proteins in the vascular endothelial cells of the AD brain, leading to activation of microglia.

## Introduction

Blood brain barrier (BBB) protects the brain parenchyma from circulating toxins, immune cells, and pathogens while regulating the transport of essential nutrients. BBB is vital to homeostasis of the brain, and with aging its integrity begins to decrease. An MRI study has provided direct evidence to show BBB breakdown in the aging human brain (Montagne et al., 2015). Therefore, BBB breakdown is also associated with aging-associated neurodegenerative diseases (Sweeney et al., 2019). BBB dysfunction is reported to be both cause and consequences of Alzheimer’s disease (AD) (Erickson and Banks, 2013). Two-hit vascular hypothesis suggests that a primary cerebrovascular dysfunction (first hit) can cause BBB damage-induced defective Aβ clearance (second hit), leading to the neurodegenerative cascade (Zlokovic, 2005). The breakdown of BBB has been observed in 5XFAD mice, a transgenic Alzheimer’s mouse model with amyloid pathology, at 4 months of age and it was shown to be mimicked in a cell culture model with cerebral endothelial cells (Liu et al., 2020). Previous studies have reported that consumption of calorie-rich western diet leads to compromise BBB integrity (Kanoski et al., 2010; Hsu and Kanoski, 2014; Hargrave et al., 2016). Western diet consists of saturated fat and simple sugars. The effect of these components on BBB disruption and associated biological effects in brain microvascular endothelial cells (BMECs) remained mostly unidentified.

Blood brain barrier consists of BMECs, end feet of astrocytes, and pericytes, collectively known as neurovascular unit (NVU) (Iadecola, 2017). Unlike other vascular endothelial cells, BMECs are glued together by tight-junction (TJ) proteins (e.g., occludins and claudins) and scaffolding proteins- zona occludens (e.g., ZO-1 and ZO-2) (Haseloff et al., 2015). Particularly, claudins are essential to guard the barrier functions, as loss of them increases barrier permeability. While, scaffolding proteins (ZO-1 and ZO-2) are important for binding claudins to the TJs (Hartsock and Nelson, 2008; Daneman and Prat, 2015). BMECs are uniquely positioned at the interface between peripheral blood circulation and the central nervous system (CNS). Circulating factors in aged mice have been shown to induce inflammatory mediators through upregulation of vascular cell adhesion molecule 1 (VCAM1) and intercellular adhesion molecule-1 (ICAM1) (Su et al., 2012; Yousef et al., 2019). Increased inflammation reduced TJ proteins namely claudin-5, ZO1 and occludins. Further, BBB breakdown caused microglial reactivity, neuroinflammation, and cognitive deficits in aged mice, which were counteracted by systemic administration of anti-VCAM-1 antibody or by genetic silencing of VCAM1.

Alzheimer’s disease often coexists with other brain lesions caused by comorbidities which can exert their deleterious CNS effects through NVU. The precondition for the comorbidities is called metabolic syndrome (MetS), a highly prevalent condition among adult population (Aguilar et al., 2015). MetS can be caused by the downregulation of silence information regulator 3 (SIRT3), a deacetylase enzyme that enhances the function of key mitochondrial proteins (Hirschey et al., 2011). We have reported that deletion of Sirt3 gene leads to downregulation of metabolic enzymes, mitochondrial dysfunction and neuroinflammation in the brain, the main causes of Alzheimer’s disease (Tyagi et al., 2018). In a subsequent recent publication, we reported that SIRT3 deficiency in AD mice exacerbates brain insulin resistance, neuroinflammation, amyloid plaque deposition and proliferation of glial cells (Tyagi et al., 2020). Because 20% of total energy consumption is in the brain, it is highly vascularized to facilitate the uptake of oxygen and nutrients. Studies have revealed that to meet the high energy requirement for active transport across BBB, endothelial cells contain a large number of mitochondria, and they are susceptible to oxidative stress (Navaratna et al., 2013). BMECs also have high metabolic activity because of their active transport function. Therefore, under conditions of metabolic dysregulation as observed during diet-induced obesity, BMECs can be susceptible to injury. A study, by Hargrave et al. (2016) showed that diet-induced obesity in rats leads to BBB leakage in multiple regions of hippocampus along with cognitive dysfunction.

Loss of BBB integrity can lead to dysregulation of microglia, the immune cells of the brain. Microglia in their entire life span, do not directly encounter the systemic circulation because of the BBB (Crotti and Ransohoff, 2016). Although microglial regulation is primarily by brain intrinsic events, changes in the periphery are known to modulate microglial behavior (Dilger and Johnson, 2008). Increased BBB permeability can lead to fibrinogen infiltration and microglial activation (Ryu and McLarnon, 2009). Dual opposing effects of microglia have been demonstrated during systemic inflammation by *in vivo* imaging (Haruwaka et al., 2019). In this study, microglia were shown to migrate to the cerebral vasculature and enhance BBB integrity by increasing the expression of claudin-5 in response to systemic inflammation. However, during sustained inflammation, microglia phagocytosed astrocytic end-feet and cause BBB injury.

Thus, findings from previous studies suggest that changes at the systemic level can affect the internal milieu of the brain when BBB permeability is increased. We hypothesized that comorbidities may exacerbate Alzheimer’s pathogenesis by a mechanism involving BBB, specifically BMECs. Therefore, the objective of this study was to determine how the circulating factors in comorbidities act at the level of BBB and regulate microglia.

## Materials and Methods

### AD Post-mortem Samples

Post-mortem brain samples of 70–90-year-old AD cases and controls were obtained from Garrison Institute of Aging, Lubbock, TX, United States. Both groups were age and sex matched. The Alzheimer’s cases were at the Braak stage of 5 or 6 and these brain samples were positive for amyloid plaques and neurofibrillary tangles while they were absent in control group. The post-mortem intervals were between 2 and 5 h for all brain samples used for the study.

### Western Diet Feeding of APP/PS1 Mice

Animal care and the experimental procedures were approved by IACUC at Rocky Mountain Regional Veteran Affairs Medical Center, Aurora CO, United States. C57BL/6 (wild type; WT) and APP/PS1 (AD mice, stock # 5864) mice in C57BL/6 genetic background were obtained from Jackson Laboratory (Bar Harbor, ME, United States). To generate the WT and AD mice for experimentation, female C57BL/6 mice were crossed with male APP/PS1 mice. Tail snips from each offspring were subjected to genotyping for PSEN1. Six-weeks old male mice (WT and AD, 6/group) were fed *ad libitum*, a standard diet (TD.2018 Envigo, Indianapolis, IN, United States) or a calorie rich western diet (TD.08811, Envigo, Indianapolis, IN, United States) for 7 months. Western diet contained 17.3% protein, 47.6% carbohydrate and 23.2% fat by weight which generate 14.7% kcal, 40.7% kcal, and 44.6% kcal, respectively. While composition of standard diet was 18.6% protein (24% kcal), 44.2% carbohydrate (58% kcal) and 6.2% fat (18% kcal). At the end of the study, mice were subjected to CO2 asphyxiation followed by euthanasia. Blood was collected by cardiac puncture in BD Microtainer tubes coated with K2 EDTA followed by centrifugation at 4°C for 15 min at 2000 rpm, and separated plasma was stored at – 80°C for analysis. The brain was snap frozen in liquid nitrogen and stored at – 80°C for RNA and protein analyses.

### Cell Cultures

Mouse Brain Endothelial Cells (BEND3) and Human Brain Microvascular Endothelial Cells (HBMECs) were obtained from American Type Culture Collection (Cat # CRL-2299, Manassas, VA, United States) and NEUROMICS (Cat # HEC02, Edina, MN, United States), respectively. BV2 cells, mouse microglial cell line, cells were a kind gift from Dr. Dennis Selkoe (Harvard Medical School, Boston, MA, United States). BEND3 and BV2 cells were cultured in advanced DMEM/F12 medium (Cat # 12634-010, Gibco, Gaithersburg, MD, United States) containing 10% fetal bovine serum and 1% penicillin-streptomycin under standard culture conditions, and Endo-Growth Media (Cat # MED001, NEUROMICS, Edina, MN, United States) was used to grow HBMECs. **Generation of Sirt3-silenced cell lines:** BEND3 and BV2 cells were infected with Sirt3 shRNA Lentiviral particles (Cat # sc 61556) or control shRNA Lentiviral particles (Cat # sc 108080) using polybrene (0.5 μg/ml; Cat # sc 134220). Stable infected cells were selected using puromycin dihydrochloride (8 μg/ml, Cat # sc 10807) in DMEM media supplemented with 10% FBS for 4 weeks, and cells were pooled and maintained in the same medium. All the reagents, used for transfection, were from Santa Cruz Biotechnology (Dallas, TX, United States). Silencing of Sirt3 gene was confirmed by western blot analysis in stable infected cells.

### RNA Isolation, PCR Array and RT-PCR Analysis

Total RNA was isolated from BEND3 and Sirt3-silenced (shSirt3) BEND3 cells exposed to high glucose and palmitic acid, using Versagene RNA isolation kit (Thermo Fisher Scientific, Hampton, NH, United States). RNA samples were treated with DNase and then converted to cDNA as described earlier (Velmurugan et al., 2012). The cocktail for PCR array was prepared by adding 1278 μl of the RT^2^ qPCR SYBR Green master mix and 1173 μl H_2_0 to 102 μl of the diluted cDNA and 25 μl of this cocktail was added to each well of the 96-well PCR array plate (SABiosciences, Frederick, MD, United States) containing primers for the 84 genes in mouse inflammatory pathway, 5 housekeeping control genes and 3 RNA and PCR quality controls as in our previous study (Qin et al., 2016). The mRNA levels of Cox-2, CXCL1 RANTES, and GADD45β were measured by real-time quantitative RT-PCR using Taqman probes. Amplicons corresponding the amplification sequence was synthesized and used as standards in the RT-PCR analysis and the mRNA levels of chemokines were expressed in attograms (ag) per pg of GAPDH.

### Western Blot Analysis

Cell/tissue lysates, from cultured cells, frontal cortical tissue of mice, and post-mortem human brain of AD patients and age-matched controls, were prepared with mammalian protein extraction buffer (Pierce, Rockford, IL, United States), supplemented with phosphatase and protease inhibitors as described previously (Pugazhenthi et al., 2013; Tyagi et al., 2018). The total protein concentration was determined by using Bradford assay Kit (Cat # 5000001, Bio-Rad, Hercules, CA, United States) in the supernatant of cell/tissue lysates. Briefly, equal amount of protein (∼30–50 μg/well) was resolved on a gradient 4–20% SDS-PAGE and separated proteins were then transferred to PVDF membrane. The membranes were blocked in blocking buffer (5% non-fat milk in TBST) for 1 h at room temperature. Subsequently, membranes were incubated with primary antibodies (1:1000) overnight on shaker at 4°C, followed by alkaline phosphatase conjugated appropriate secondary antibody for 1 h. Signals were visualized by CDP-star reagent (Sigma Aldrich-St Louis, MO, United States) using ChemiDoc Imaging System (Bio-Rad, Hercules, CA, United States). The band intensities were quantified with reference to β-actin control bands, using Image Lab software from Bio-Rad. Primary antibodies, SIRT1 (Cat # 9475), SIRT3 (Cat # 5490), SIRT5 (Cat # 8782), SIRT6 (Cat # 12486), SIRT7 (Cat # 5360), Cox-2 (Cat # 12282), IKB-α (Cat # 4814), NF-κB p65 (Cat # 8242), Matrix metallopeptidase 9 (MMP-9; Cat # 13667) and β-actin (Cat # 4967) from Cell Signaling Technology (Danvers, MA, United States); claudin-5 (Cat # ab15106), Occludin (Cat # ab167161), ZO-1 (Cat # ab59720), CXCL1 (Cat # ab86436) and RANTES (Cat # ab189841) from AbCam (Cambridge, MA, United States); GADD45β (Cat # MBS821452) from MyBioSource (San Diego, CA, United States), were used for immunoblotting.

### Immunofluorescent Staining

Cells were fixed in 4% paraformaldehyde for 20 min followed by gentle rinsing three times for 5 min each with PBS. The cells were permeabilized with 5% BSA and 0.2% Triton X-100 in PBS for 60 min and then incubated with primary antibodies, rabbit anti-claudin-5 (Cat # ab15106; 1:1000 dilution), rabbit anti-ZO-1 (Cat # ab59720; 1:1000 dilution) or rabbit anti-Iba1 (Cat # 019-19741, Wako, Richmond, VA, United States;1:2000 dilution), overnight at 4°C in a humidified chamber. Next day, following three washes with PBS, cells were incubated with appropriate secondary antibodies conjugated to Alexa Fluor (Alexa Flour 488 or Alexa flour 594) and DAPI (2 μg/ml; nuclear stain) in dark for 1 h followed by three washes with PBS. The immuno-stained cells were mounted with Prolong Gold Antifade reagent, and images of stained cells were captured with the Leica SP8 confocal microscope with white laser, using a Leica HC PL APO 40 × 1.30 NA oil objective.

### Microglial - Endothelial Cocultures and TEER Measurement

Transendothelial electrical resistance (TEER) is an important measure of BBB integrity, using a cell culture model. To determine how the interactions between microglia and BMEC lead to compromised BBB integrity, and further to identify how silencing Sirt3 gene in these cells affects BBB, thus these cells were cocultured as shown in Figure 6A. BV2 or shSirt3-BV2 cells were cultured on the bottom well of a 6-well dish. In parallel, BEND3 or shSirt3-BEND3 cells were cultured on coated transwell inserts, placed in another 6-well dish. When the cell culture reached ∼70% confluence, the inserts were placed in over cultured microglia. First, the inserts were placed in the dish without microglia to measure background resistance at 37°C with heating plate using EVOM resistance meter (World Precision Instrument, Sarasota, FL, United States). Then the inserts with endothelial cells were placed in dishes with microglia and treated with high glucose (30 mM), palmitic acid (300 μM) or the combination of both for 48 h. In another set of similar experiment, we exposed BV2 cells to a combination of cytokines (20 ng/ml TNF-α, 20 ng/ml IL-1β, and 20 ng/ml IFNγ) and the coculture was continued for 6 days. Resistance was measured at 24 h time intervals. Altered endothelial cell permeability was determined from the TEER values.

### Plasma Assays

Insulin levels were determined using mouse Insulin ELISA kit (Cat # 80-INSMS-EO1, ALPCO, Salem, NH, United States). C-reactive protein (CRP) and interleukin-1beta (IL-1β) were measured using CRP (Cat # MCRP00) and IL-1β (Cat # MLB00C) mouse ELISA kit, respectively, from R &D systems (Minneapolis, MN, United States). Plasma triglycerides were assayed using a colorimetric kit (Cat # ab65336) from Abcam, Cambridge, MA, United States. Assays were performed following the manufacture’s protocol.

### Statistical Analysis

All statistical analyses were carried out using GraphPad software. Significant differences between groups were determined by one-way ANOVA followed by Dunnett’s test, and two-sided *P* values of < 0.05 were considered significant.

## Results

### Western Diet Induces Insulin Resistance and Neuroinflammation and Reduces the BBB Integrity in APP/PS1 Mice

**FIGURE 1:**
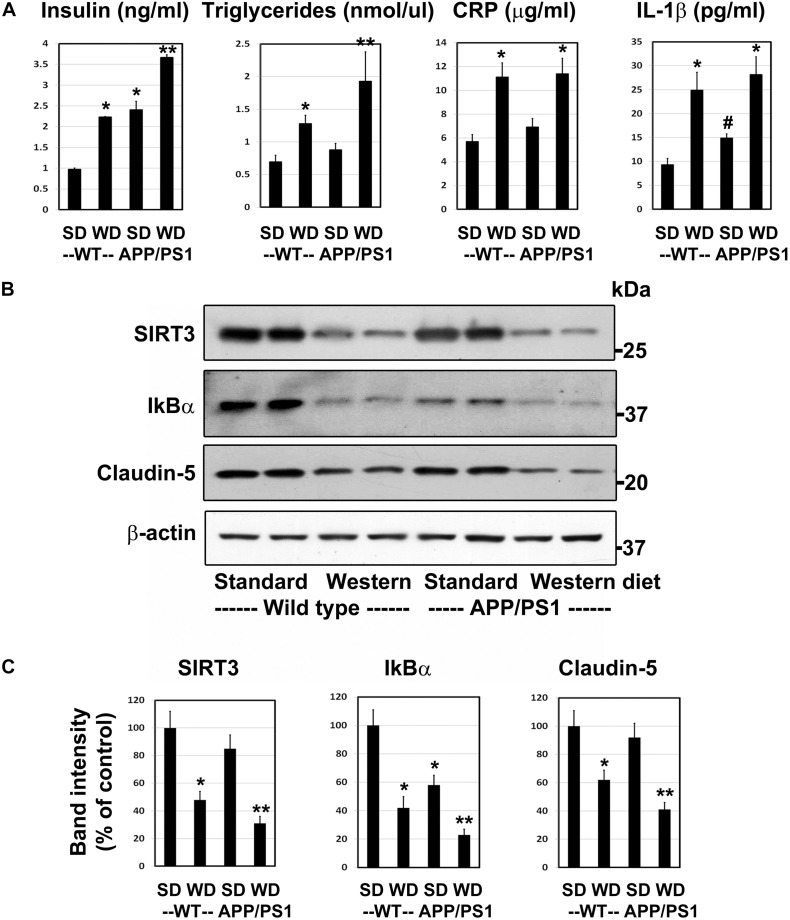
Western diet induces insulin resistance and neuroinflammation and reduces the BBB integrity in APP/PS1 mice. **(A)** 6 weeks-old Wild type (WT) and APP/PS1 male mice were fed on standard diet (SD) or energy-rich western diet (WD) for 7 months. Plasma levels of insulin, CRP and IL-1β were assayed by ELISA. Plasma triglycerides levels were determined calorimetrically using assay kit. All assays were performed following the manufactures’ protocol. **(B)** Mouse cortical samples from the four groups of mice were collected for the Western blot analysis of SIRT3, IkBα and claudin-5. The blots were re-probed for β-actin. **(C)** The band intensities were determined by scanning, corrected for β-actin levels, and expressed as percent of control. Data are expressed as mean of ± SE (*n* = 6) for each group. ^#^, *P* < 0.05; *, *P* < 0.01; **, *P* < 0.001 vs WT mice on standard diet.

Having characterized a genetic model for comorbid AD previously (Tyagi et al., 2020), herein we examined a lifestyle-based AD model with metabolic syndrome (MetS). As expected, western diet feeding in wild type mice resulted in 2–2.7-fold increase (*P* < 0.01) in plasma levels of insulin, triglycerides, CRP and IL-1β (Figure 1A). APP/PS1 mice were also characterized by significant hyperinsulinemia (*P* < 0.01) and modest increases in the levels of IL-1β (*P* < 0.05). The combination of western diet and amyloid pathology resulted in significant increases in the circulating levels of insulin (3.8-fold, *P* < 0.001), triglycerides (2.8-fold, *P* < 0.001), CRP (2.0-fold, *P* < 0.01), and IL-1β (3.0-fold, *P* < 0.01) as compared to wild type mice fed standard diet (Figure 1A), suggesting diet-induced exacerbation of insulin resistance and inflammation. Further examination of the brain frontal cortical samples by Western blot analysis (Figure 1B), followed by quantitation (Figure 1C) revealed 52% (*P* < 0.01) decrease SIRT3 levels in the western diet-fed wild type mice. IkBα levels decreased by 58%, (*P* < 0.01) suggesting NF-kB activation. BMECs are an important component of BBB. The uniqueness of BMECs is due to TJs proteins, including claudin-5. There was a 38% decrease (*P* < 0.01) in claudin-5 levels in western diet-fed mice. SIRT3 and claudin-5 did not change significantly in APP/PS1 mouse brain whereas they were significantly reduced in western-diet-fed APP/PS1 mouse brain, compared to wild type mouse brain. However, IkBα expression was significantly less (42%, *P* < 0.01) in APP/PS1 mice, and further its levels decreased by 77% (*P* < 0.001) in western diet-fed APP/PS1 mice, as compared to wild type control mice. Overall, western diet-fed APP/PS1 mice were characterized by hyperinsulinemia, and neuroinflammation like our previously reported genetic model of comorbid AD (Tyagi et al., 2020). In addition, western diet reduced the levels of claudin-5, suggesting compromised BBB integrity.

### Downregulation of Sirtuin Pathway and Compromised BBB Integrity in the Human Alzheimer Post-mortem Brain

**FIGURE 2:**
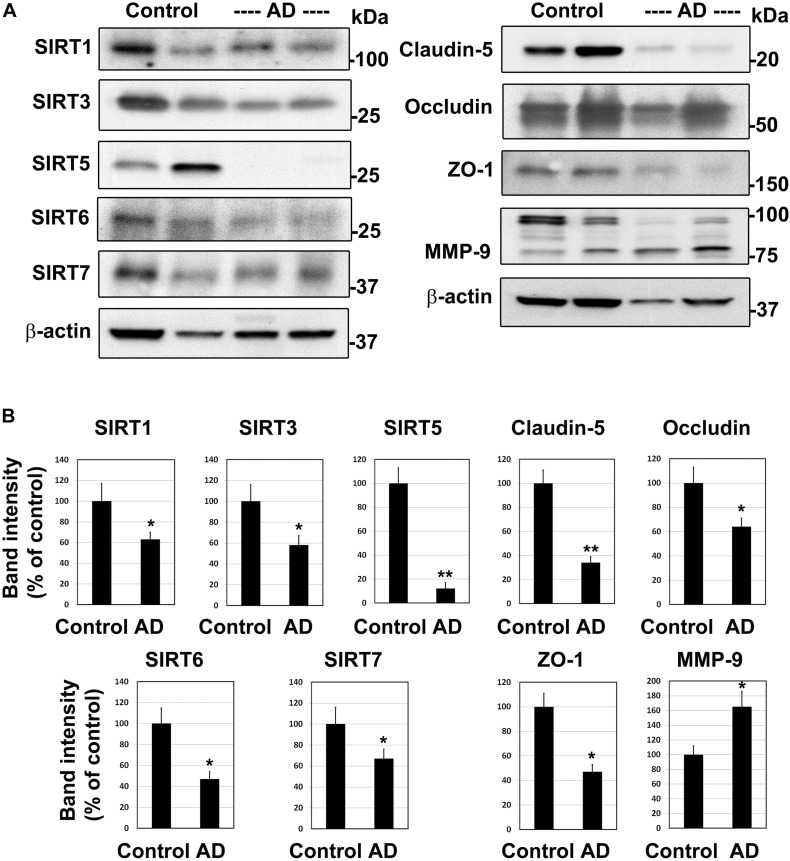
Downregulation of sirtuin pathway and compromised BBB integrity in the human Alzheimer post-mortem brain. **(A)** Post-mortem brain samples of AD cases and age-matched controls were obtained from Garrison Institute of Aging, Lubbock, TX. Western blot analysis was performed for SIRT1, SIRT3, SIRT5, SIRT6, SIRT7, MMP-9 and tight junction proteins, claudin-5, occludin and ZO-1. The blots were re-probed for β-actin. Representative images of 20 samples are presented. **(B)** The band intensities were determined by scanning, corrected for the levels of β-actin and expressed as percent of control. *, *P* < 0.01; **, *P* < 0.001 vs control. AD, Alzheimer’s disease.

To determine if the findings in mouse models of comorbid AD are observed in human AD, we examined post-mortem human brain samples of AD cases and age-matched controls by Western blot analysis (Figure 2A). The sirtuin family of proteins were significantly reduced in Alzheimer’s patients compared to age-matched controls (Figure 2B). The levels of SIRT1, 3, 6, and 7 decreased by 33–53% (*P* < 0.01). SIRT5 was nearly absent (88%, *P* < 0.001) in AD brain samples. These findings are like our observations in APP/PS1/Sirt3^–/–^ mouse brain (Tyagi et al., 2020). Damage to BBB is another well-known feature of AD brain (Erickson and Banks, 2013; Sweeney et al., 2019). As expected, the levels of TJ proteins claudin-5, occludin and ZO-1 decreased by 36–66% (*P* < 0.01-*P* < 0.001), suggesting compromised BBB integrity in the AD brain. MMP-9 is an enzyme that belongs to zinc metalloproteinases family which plays a key role in the degradation of the extracellular matrix of BBB (Van Dyken and Lacoste, 2018). The levels of the active cleaved MMP-9 (lower band) increased by 66% (*P* < 0.01) in the Alzheimer’s brain. This enzyme may play an additional role in increasing BBB permeability in this neurodegenerative disease. These two sets of findings on sirtuins, involved in metabolic regulation and TJ proteins, markers of BBB integrity raise a key question of whether they are causally linked. Therefore, we used cell culture studies to address the link between these two pathways.

### Glucolipotoxicity-Mediated BMEC Injury

**FIGURE 3:**
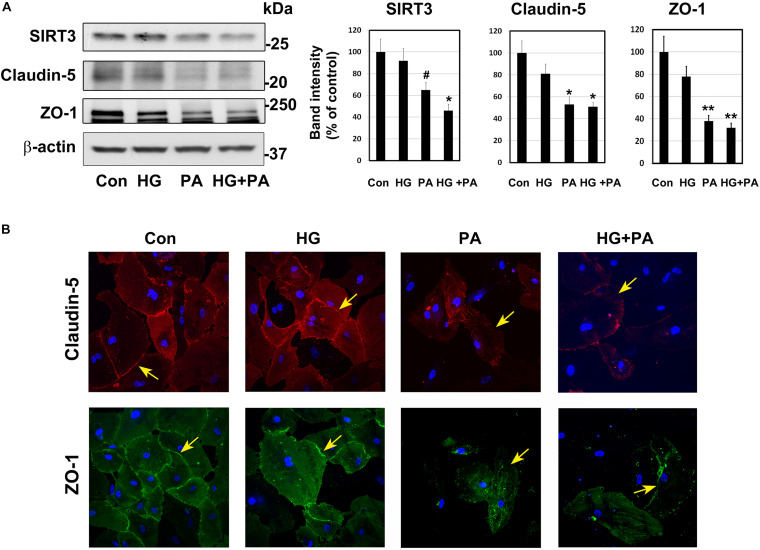
Glucolipotoxicity-mediated BMEC injury. **(A)** BEND3 cells, a mouse brain endothelial cell line, were exposed to HG, PA or the combination of both for 48 h and processed for the immunoblotting of SIRT3, claudin-5 and ZO-1. The blots were re-probed for β-actin. The bands were scanned and corrected for the levels of β-actin. ^#^, *P* < 0.05; *, *P* < 0.01; **, *P* < 0.001 compared to untreated controls. **(B)** BMECs isolated from human fetal brain (Neuromics) were cultured in the presence of HG, PA or combination of both for 24 h. The cells were fixed and immunostained for claudin-5 with Alexa flour 594 (red; upper panel) or for ZO-1 with Alexa flour 488 (green; lower panel). The nuclei were stained blue with DAPI. Images of stained cells were captured with the Leica SP8 confocal microscope with white laser, using a Leica HC PL APO 40 × 1.30 NA oil objective. HG, high glucose (30 mM); PA, palmitic acid (300 μm).

Under conditions of MetS, endothelial cells are constantly exposed to high glucose and dyslipidemia, resulting in vascular injury. To determine the effects of glucolipotoxicity on BMECs, we performed cell culture studies with BEND3 cells, a mouse brain microvascular endothelial cell line. Following exposure of these cells to high glucose (30 mM) and palmitic acid (300 μM), we examined the levels of SIRT3 and TJ proteins by Western blot analysis. Palmitic acid decreased the levels of SIRT3 (35%; *P* < 0.05), claudin-5 (47%; *P* < 0.01) and ZO-1 (62%; *P* < 0.001) (Figure 3A). Surprisingly, high glucose did not change their levels. With the combination of high glucose and palmitic acid, the decreases in SIRT3 and TJ proteins were by 50–68% (*P* < 0.01-*P* < 0.001). Overall, palmitic acid was found to be more toxic to BEND3 cells compared to high glucose. In addition, we exposed primary human BMECs to high glucose and palmitic acid and examined the TJ proteins by immunofluorescent staining. We observed significant loss of claudin-5 (upper panel) and ZO-1 (lower panel) when the endothelial cells were exposed to palmitic acid (Figure 3B). High glucose did not have any effect on TJ proteins.

### Induction of Inflammatory Genes in Endothelial Cells Exposed to Glucolipotoxicity

**FIGURE 4:**
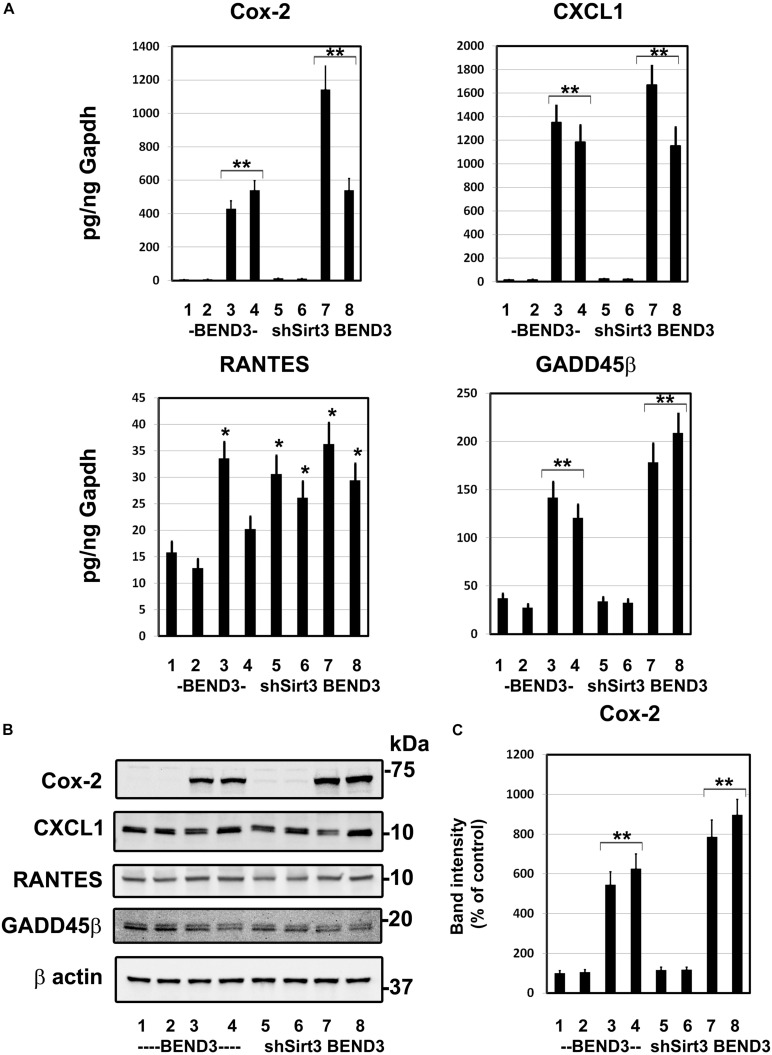
Induction of inflammatory genes in endothelial cells exposed to glucolipotoxicity. **(A)** BEND3 and shSirt3- BEND3 cells were cultured in the absence (1) or presence of 30 mM glucose (2), 300 μM palmitic acid (3) or combination of both (4) for 48 h. The cells were processed for RNA isolation and RT-PCR analysis of Cox-2, CXCL1, RANTES, and GADD45β. mRNA levels of chemokines were expressed in attograms (ag) per pg of GAPDH. **(B)** BEND3 and shSirt3-BEND3 cells were cultured in the absence (1) or presence of 30 mM glucose (2), 300 μM palmitic acid (3) or combination of both (4) for 48 h. The cell lysates were prepared and immunoblotted for Cox-2, CXCL1, RANTES and GADD45β. The blots were re-probed for β-actin. **(C)** The bands for Cox-2 were scanned and corrected for the levels of β-actin. *, *P* < 0.01; **, *P* < 0.001 vs untreated controls. shSirt3, Sirt3-silenced.

To determine if BMECs stressed in MetS release inflammatory mediators in the brain, we exposed BEND3 and shSirt3-BEND3 cells to a combination of high glucose and palmitic acid. Subsequently performed pathway-specific array for NF-kB target genes with these treated cells. We observed strong induction of several genes in the inflammatory pathway following exposure to high glucose and palmitic acid (Table 1). There was a 124-fold induction of Cox-2 gene and 28-fold induction of CXCL1 in BMECs exposed to a combination of high glucose and palmitic acid. Other chemokines including RANTES (CCL5), GADD455β and CXCL10 were also induced significantly. In general, the induction was significantly more with Sirt3-silenced endothelial cells, suggesting that MetS exacerbates the inflammatory response. To further confirm the induction of selected inflammatory genes, we performed quantitative RT-PCR analysis, using Taqman probes. For this experiment, the cells were treated with high glucose, palmitic acid or the combination of them. Interestingly, the induction was observed only with palmitic acid and not with glucose (Figure 4A). Cox-2 was induced by 96 and 204 folds (*P* < 0.001) in BEND3 and shSirt3-BEND3 cells, respectively. Similar induction of CXCL1 by the fatty acid was observed in these cells. Exacerbated induction in Sirt3-silenced BEND3 cells suggest that these cells are more prone for inflammatory response under conditions of SIRT3 deficiency which will be seen in MetS. The mRNA levels of RANTES and GADD45β were also elevated significantly (*P* < 0.01-*P* < 0.001), following exposure to palmitic acid although to a smaller extent. Next, we examine the levels of induced inflammatory mediators at the protein levels, we performed Western blot analysis with BEND3/shSirt3-BEND3 cells incubated in the presence of high glucose, palmitic acid or in combination of both (Figure 4B). Fivefold increase in the levels of Cox-2 protein was observed following exposure to palmitic acid to BEND3 cells (*P* < 0.001). In the case of shSirt3-BEND3 cells, palmitic acid increased Cox-2 levels by eightfolds (*P* < 0.001, Figure 4C). Surprisingly, in the case of other inflammatory mediators, CXCL1, RANTES, and GADD45β, we did not observe any significant change at the protein level, suggesting impairment of translation or stability of their mRNA.

**TABLE 1:** BEND3 and shSirt3-BEND3 cells were incubated in the absence (control) and presence of a combination of high glucose (30 mM) and palmitic acid (300 μM) for 48 h.

	PCR array of NF-κB target genes			
	
	————BEND3————		————shSirt3-BEND3————	
		
	Control	HG + PA	Control	HG + PA
Cox-2	1	124	2.4	256
CXCL1	1	27.9	1.01	39.9
RANTES	1	6.1	1.8	15
GADD45β	1	6.8	−1.14	10.9
Adrenomedullin	1	7.4	−1.04	10.9
CXCL10	1	2.2	1.2	5.5
NQO1	1	3.0	1.6	3.1
Myc	1	2.6	−1.05	2.4
NF-κBIA	1	2.3	1.2	2.3

*The cells were processed for RNA isolation and pathway-specific gene expression array was performed. Fold inductions over BEND3-control are presented. shSirt3, Sirt3-silenced.*

### Effects of SIRT3 Deficiency on Microglial and Endothelial Cell Interactions

**FIGURE 5:**
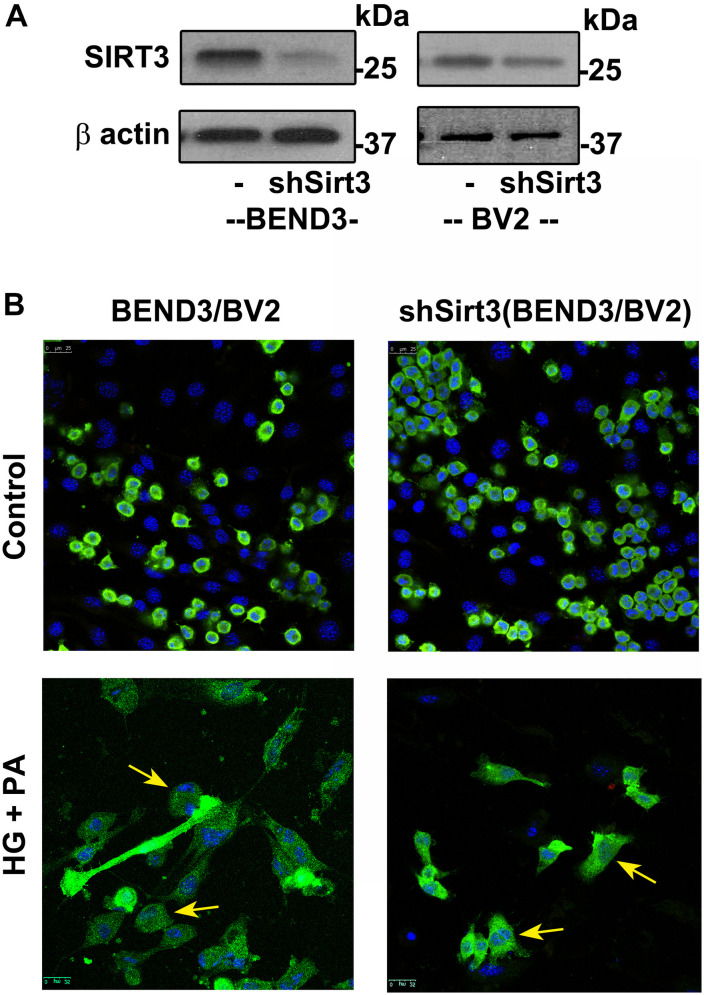
Effects of SIRT3 deficiency on microglial and endothelial cell interactions. **(A)** Lysates, from BEND3, shSirt3-BEND3, BV2, and shSirt3-BV2 cells, were immunoblotted for SIRT3 and β-actin. **(B)** Mixed cultures of BEND3/BV2 and shSirt3-BEND3/shSirt3-BV2 were incubated in the absence and presence of high glucose (HG 30 mM) and palmitic acid (PA 300 μm) for 48 h. The treated cells were fixed and immunostained for Iba1 with Alexa Flour 488 (green). Nuclei were stained blue with DAPI. Images of stained cells were captured with the Leica SP8 confocal microscope with white laser, using a Leica HC PL APO 40 × 1.30 NA oil objective. shSirt3, Sirt3-silenced.

To determine if SIRT3 deficiency dysregulates interactions between microglia and endothelial cells in the presence of high glucose and palmitic acid, we performed mixed cultures of BV2/BEND3 and shSirt3-BV2/shSirt3-BEND3 cell lines. Western blot analysis showed significant reduction (∼ 75%) in SIRT3 protein levels in the shSirt3-BEND3 and shSirt3-BV2 stable cell lines (Figure 5A). Immunofluorescent staining for microglial cells in the mixed culture showed changes in microglial morphology, suggesting activation, following exposure to a combination of high glucose and palmitic acid (Figure 5B). In the case of mixed cultures with Sirt3-silenced cell lines, glucolipotoxicity was more along with enhanced microglial activation, suggesting that microglial and endothelial interactions may be dysregulated during MetS.

**FIGURE 6:**
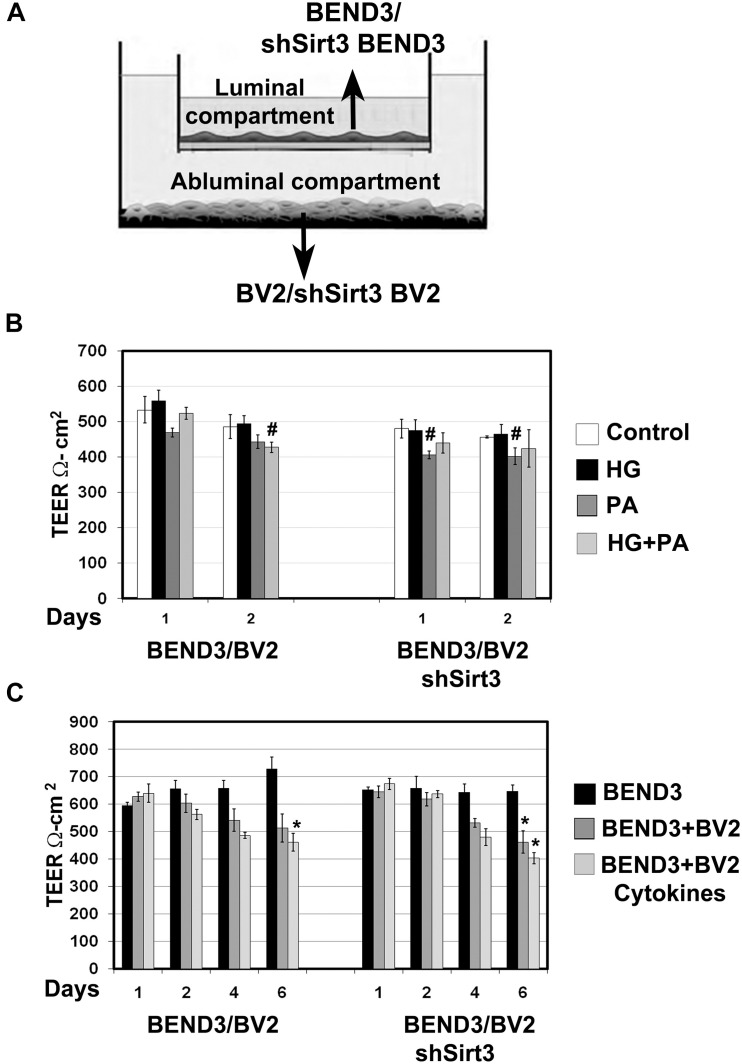
Measurement of barrier function in endothelial cells (TEER). **(A)** An *In vitro* co-culture model of microglial and endothelial cells: Culture of BEND3 or shSirt3-BEND3 cells in the Boyden chamber inserts, while BV2 or shSirt3-BV2 cells in the bottom wells were performed. **(B)** The cocultures were incubated in the absence and presence of high glucose (HG 30 mM), palmitic acid (PA 300 μm), or combination of both for 24 h and 48 h. TEER was measured, using EVOM resistance meter after 24 h and 48 h. **(C)** The cocultures were exposed to a combination of cytokines (20 ng/ml each of TNF-α, IL-1β, and IFN-γ) for 6 days, followed by TEER measurement. ^#^, *P* < 0.05; *, *P* < 0.01 vs untreated controls. shSirt3, Sirt3-silenced.

### Measurement of Barrier Function in Endothelial Cells (TEER)

Brain microvascular endothelial cells are unique compared to other endothelial cells because they are joined together by TJ proteins which play a key role in BBB integrity. TEER measurement is a standard method to determine electrical resistance across BMECs. To determine if interactions between BMECs and microglia affects BBB permeability and if SIRT3 plays a role in this interaction, we performed coculture of BEND3 and BV2 cells as shown in Figure 6A. Similar coculture was also performed with shSirt3-BEND3 and shSirt3-BV2 cells. When these cocultures were exposed to palmitic acid, there was a modest decreases (*P* < 0.05) in TEER values (Figure 6B). Interestingly, TEER values were slightly higher (not significant) when cells were exposed to high glucose + palmitic acid, suggesting high glucose might be protecting the cells from lipotoxicity effect of palmitic acid (Figure 6B). Similar results were observed with coculture of shSirt3-BEND3 and shSirt3-BV2 cells. Because there was toxicity to the cells with palmitic acid after 48 h, we designed another long-term exposure with a combination of cytokines, as a model for chronic inflammation. We observed that permeability across BMECs increased (decreased TEER) in a time-dependent manner when cocultured with microglial cells, exposed to a mixture of cytokines (Figure 6C). The decrease was more when Sirt3-silenced cells (BV2 and BMECs) were used for coculture.

## Discussion

**FIGURE 7:**
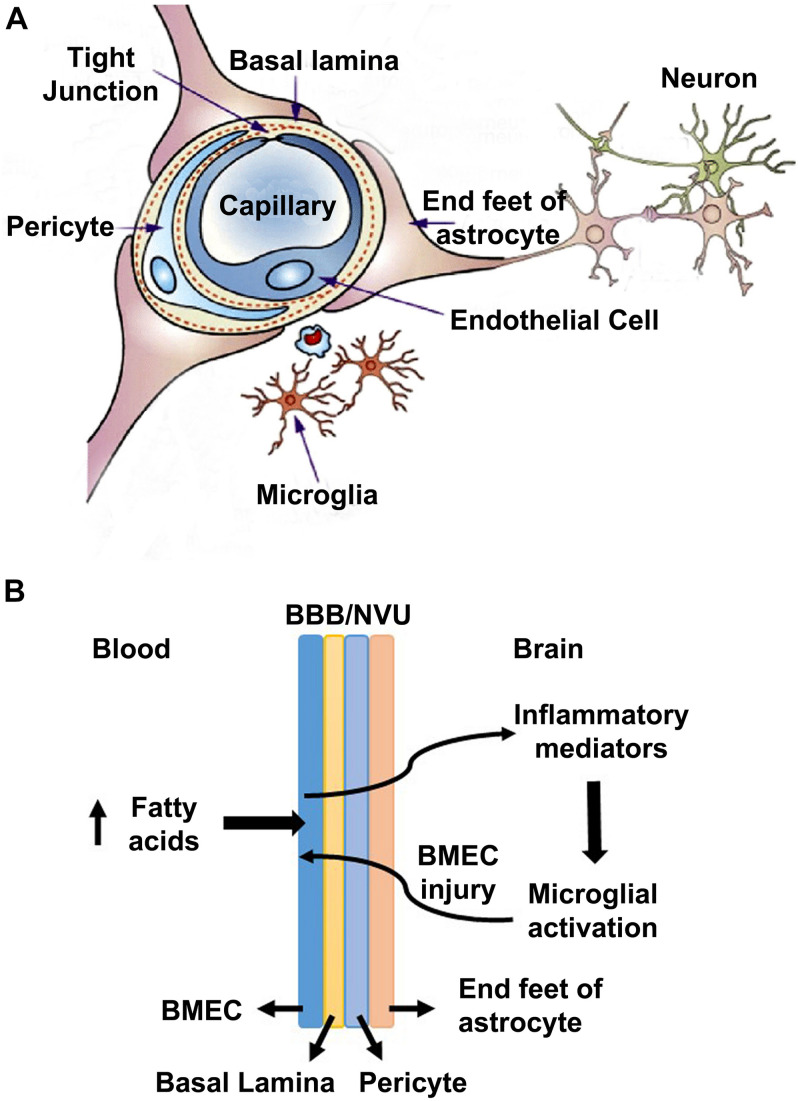
A model for the interactions of obesity with BBB **(A)** A schematic diagram for the neurovascular unit (NVU) is presented here. NVU comprises of brain microvascular endothelial cells (BMECs), end feet of astrocytes and pericytes. BMECs are glued together by tight-junction (TJ) proteins, which form the blood brain barrier to maintain the homeostasis of the brain. **(B)** A potential mechanism is presented here, based on the findings of this study. High concentration of saturated fatty acids in blood, as observed in obesity, acts on BMECs and induces the expression of inflammatory mediators, which activate microglia. Activated microglia, in turn acts on BMECs, leading to a vicious cycle.

Several prior studies have suggested that the pure form of AD is rare, and it coexists with the brain lesions caused by comorbidities namely obesity, diabetes, hypertension, and cardiovascular disease (Montine et al., 2012; Kawas et al., 2015; Baglietto-Vargas et al., 2016; White et al., 2016). In a recent study, we reported the generation of a genetically induced comorbid AD mouse model with amyloid pathology and MetS (Tyagi et al., 2020). In this comorbid mouse model, exacerbation of MetS, neuroinflammation, and Alzheimer’s pathology were observed. MetS, a cluster of risk factors, is the early phase of comorbidities. There are multiple pathways through which MetS can interact with CNS and dysregulate homeostasis of the brain. MetS can cause vascular injury in the brain, leading to BBB breakdown, neuroinflammation and microglial activation (Newcombe et al., 2018; Zlokovic et al., 2020). In the current study, we examined the effects of MetS on BMECs by *in vivo* and *in vitro* studies. Our main findings suggest the involvement of BMECs and microglia (Figure 7A). Particularly, we demonstrate that hyperlipidemia, observed in MetS and obesity modulate BMECs tight junction and induces inflammatory mediators which activate microglia, resident immune cells of the brain (Figure 7B).

Having characterized a genetic model of comorbid AD (Tyagi et al., 2020), herein we examined a life-style-based model. Following the feeding of APP/PS1 mice with western diet, enriched with saturated fat and simple sugars, we observed markers of MetS in this comorbid AD mice. These mice were characterized by hyperinsulinemia, hypertriglyceridemia and elevated levels of CRP. These findings are like our observations in APP/PS1/Sirt3^–/–^ mouse brain (Tyagi et al., 2020). Damage to BBB is another well-known feature of AD brain (Erickson and Banks, 2013; Sweeney et al., 2019). Previous studies have examined the role of BBB permeability in AD (Yamazaki et al., 2019; Huang et al., 2020) as well as during diet-induced obesity (Ouyang et al., 2014). Therefore, we examined the levels of claudin-5 (TJ protein) with brain samples and observed additive effects of AD and diet-induced obesity (Figure 1B). Similar additive effects were seen with SIRT3 levels. Examination of AD post-mortem samples revealed downregulation of sirtuin pathway and decreases in the levels of TJ proteins (Figure 2). MMP-9 is an enzyme that belongs to zinc metalloproteinases family which plays a key role in the degradation of the extracellular matrix of BBB (Van Dyken and Lacoste, 2018). We also observed elevation of the active cleaved fragment of MMP-9 which degrades TJs proteins. SIRT3 deficiency is known to accelerate the progression of MetS (Hirschey et al., 2011). Calorie restriction by fasting upregulates SIRT3 expression (Nogueiras et al., 2012). Brain vasculature is critical for cognition. Vascular dementia (VaD) is the second most common form of dementia after AD. However, significant overlap between these two forms is being recognized. The overlap ranges from AD with vascular dysfunction to mixed type of dementia (Iadecola, 2013). When cerebrovascular lesions are often observed in aged brains, it is difficult to consider VaD as a distinct type (Gorelick et al., 2011). Deteriorating vascular function and the progressive neurodegenerative process need to be viewed as converging pathogenic mechanisms.

There were elevated plasma levels of IL-1β and triglycerides in western diet-fed mice (Figure 1A). Cytokines are known to pass through BBB and induce the secondary inflammatory pathway in the brain (Banks, 2005; Yarlagadda et al., 2009). Induction of cytokines and chemokines in hippocampus is observed following systemic challenge with IL-1β and TNFα in mice (Skelly et al., 2013). Microglia are known to be primed in the aging brain and they respond to peripheral inflammation with greater severity and duration (Dilger and Johnson, 2008). Although dyslipidemia has been shown to correlate with BBB impairment in Alzheimer’s patients (Bowman et al., 2012), its effects on the CNS, especially as a potential cause of neuroinflammation has not been investigated. Our current study focused on the role of lipotoxicity observed during diet-induced obesity. In this study, we demonstrate that palmitic acid induces an array of inflammatory mediators in cultured BEND3 cells. Induction of gene expression of inflammatory mediators, including Cox-2, CXCL1, RANTES, and GADD45β were observed in these treated cells. Cox, a rate limiting enzyme in the synthesis of prostaglandins (PGs), catalyzes the conversation of arachidonic acid to PGs. While Cox-1 is constitutively expressed, Cox-2 is the inducible form. This enzyme is known to play a key role in the development of vascular inflammation (Grace et al., 2014; Voyvodic et al., 2014). Induction of Cox-2 is known to further induce inflammatory mediators in neurodegenerative diseases (Minghetti, 2004). We observed strong induction of Cox-2 at the mRNA and protein levels in BEND3 cells exposed to palmitic acid (Figure 4). Interestingly, the induction was more with Sirt3-silenced BEND3 cells, suggesting that Sirt3 deficiency exacerbates inflammatory response. Although Cox-2 mRNA levels closely mirror protein levels, surprisingly, the robust induction of other inflammatory mediators by palmitic acid was not reflected at their corresponding protein level. This discrepancy can be explained by posttranscriptional regulation of mRNA, as it is reported to vary among different cytokines/chemokines. Previous studies have suggested that the mRNA stability of cytokines and chemokines are highly regulated, and posttranscriptional regulation of their mRNA has a role in governing the inflammation (Herman and Autieri, 2017).

Previous studies have suggested that sirtuins play a role in BBB integrity. Increased BBB permeability, decreased expression of TJs protein (claudin-5) along with decreases in mRNA levels of Sirt1 in the brain microvessels have been reported in aged mice (Stamatovic et al., 2019). This study also demonstrated the loss of BBB integrity in Sirt1-silenced mice. Another study reported minocycline-mediated improvement in BBB integrity by a mechanism involving SIRT3 by *in vivo* and *in vitro* studies under hypoxic conditions (Yang et al., 2015). Minocycline decreased BBB permeability to Evans Blue in rats and increased TEER in cultured HBMECs after hypoxia. These effects were lost following the silencing of Sirt3. AMPAR antagonist perampanel has been shown to protect NVU by a mechanism involving Sirt3 (Chen et al., 2021). However, the role of sirtuins have not been examined under conditions of comorbid AD. Our findings on the dysregulation of microglial and BMEC interactions are significant in the context of SIRT3 deficiency in comorbidities which further induces an inflammatory response. We have previously reported the formation of inflammasomes in the brain samples of Sirt3^–/–^ mice (Tyagi et al., 2018).

Significant cytotoxicity and changes in microglial morphology were observed when cocultures of shSirt3-BEND3 and shSirt3-BV2 cells were exposed to HG and PA. TEER measurement with these cocultures suggested decreased barrier integrity. Previous studies have reported that microglia through their distinct phenotypes regulate BBB integrity (Ronaldson and Davis, 2020). Microglia play biphasic roles in terms of BBB integrity in a context dependent manner (Ronaldson and Davis, 2020). Following BBB injury juxtavascular microglia migrated to the site and close the leak through their processes with P2RY12 receptor (Lou et al., 2016). However, proinflammatory cytokines released from activated microglia are also known to decrease the expression of TJs and increase the expression of MMP-9 which degrades TJ proteins (Wiggins-Dohlvik et al., 2014). Perivascular microglia cause necroptosis of endothelial cells, following ischemic/reperfusion injury by a mechanism involving TNF-α (Chen et al., 2019). Aβ-stimulated microglia decrease the levels of TJ proteins in cocultured BEND3 cells whereas unstimulated microglia increased their content (Mehrabadi et al., 2017). Inflammatory signals have been shown to be amplified by communications between BMECs and glial cells (Krasnow et al., 2017). LPS-activated microglia increase the permeability of BMECs by a mechanism involving NADPH oxidase (Matsumoto et al., 2012). IL-1β induced induction of proinflammatory cytokines in microglia was significantly more when cocultured with BMECs (Zhu et al., 2019). During BBB damage, fibrinogen leakage can induce clustering of microglia near the perivascular region, leading to neuronal injury (Davalos et al., 2012).

## Conclusion

The findings of this study suggest that hyperlipidemia in comorbidities of AD can compromise BBB integrity by inducing inflammatory mediators and decreasing TJ proteins in the vascular endothelial cells of the AD brain, leading to activation of microglia. We propose a model (Figure 7) in which BMECs serve as a gateway in the crosstalk between peripheral and central inflammation. As BMECs line the luminal side, they are in constant contact with circulating factors and in communication with circulating immune cells. These cerebrovascular endothelial cells are critical sensors of peripheral inflammation and mediators of microglial activation. In this model, elevated circulating fatty acids during diet-induced obesity act on BMECs and induce inflammatory mediators which activate microglia. Under conditions of Sirt3 deficiency this microglial and endothelial interactions are further exacerbated.

## Data Availability Statement

The original contributions presented in the study are included in the article/Supplementary Material, further inquiries can be directed to the corresponding author/s.

## Ethics Statement

The animal study and the experimental procedures were approved by IACUC at Rocky Mountain Regional Veteran Affairs Medical Center, Aurora, CO.

## Author Contributions

AT and SP designed the experiments and wrote the manuscript. AT supervised the mouse experiments, sacrificed the animals, collected the tissues, and performed the immunofluorescent staining. IS did the Immunoblotting. CM contributed to the microscopic image capture and analysis. PHR provided the human post-mortem brain samples of AD cases and age-matched controls. All authors reviewed and approved the manuscript.

## Conflict of Interest

The authors declare that the research was conducted in the absence of any commercial or financial relationships that could be construed as a potential conflict of interest.

## Publisher’s Note

All claims expressed in this article are solely those of the authors and do not necessarily represent those of their affiliated organizations, or those of the publisher, the editors and the reviewers. Any product that may be evaluated in this article, or claim that may be made by its manufacturer, is not guaranteed or endorsed by the publisher.

## Abbreviations

AD: Alzheimer’s disease
BBB: blood brain barrier
BMECs: brain microvascular endothelial cells
BEND3: mouse brain-derived Endothelial cells3
Cox: cyclooxygenase
CNS: central nervous system
CRP: C-reactive protein
HG: high glucose
HBMECs: human brain microvascular endothelial cells
ICAM1: intercellular adhesion molecule-1
IL-1 β: interleukin-1beta
MetS: metabolic syndrome
MMP-9: matrix metallopeptidase 9
NF-kB: nuclear factor kappa-light-chain-enhancer of activated B
NVU: neurovascular unit
PA: palmitic acid
PGs: prostaglandins
shSirt3: Sirt3-silenced
SIRT: silence information regulator
TJ: tight junction
TEER: transendothelial electrical resistance
VaD: Vascular dementia
VCAM: vascular cell adhesion molecule
ZO: zona occludens.
