# Supplementary material for: Effects of Lipotoxicity in Brain Microvascular Endothelial Cells During Sirt3 Deficiency-Potential Role in Comorbid Alzheimer’s Disease
Source: Front Aging Neurosci. 2021 Jul 28;13:716616. doi: 10.3389/fnagi.2021.716616 (PMC8355826; doi:10.3389/fnagi.2021.716616)
Supplement: Supplementary file 1 [file Data_Sheet_1.pdf]

SIRT3

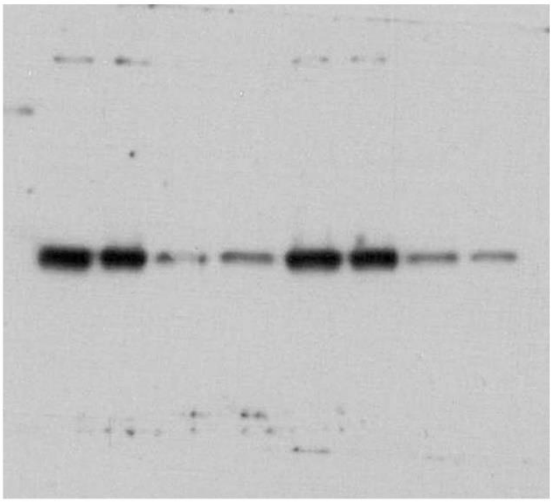

Standard Western Standard Western diet
------ Wild type ------ ----- APP/PS1 ------

IkBα

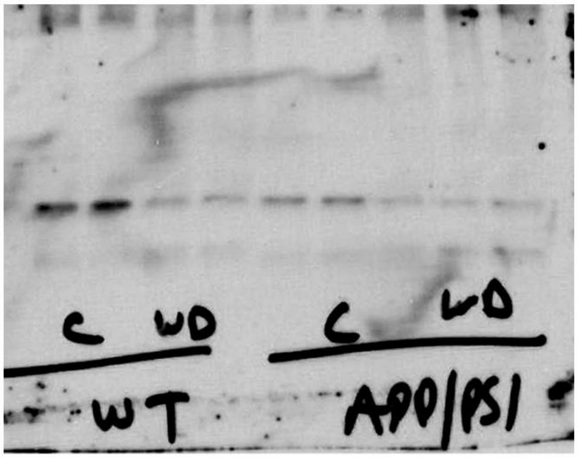

Standard Western Standard Western diet
------ Wild type ------ ----- APP/PS1 ------

Claudin-5

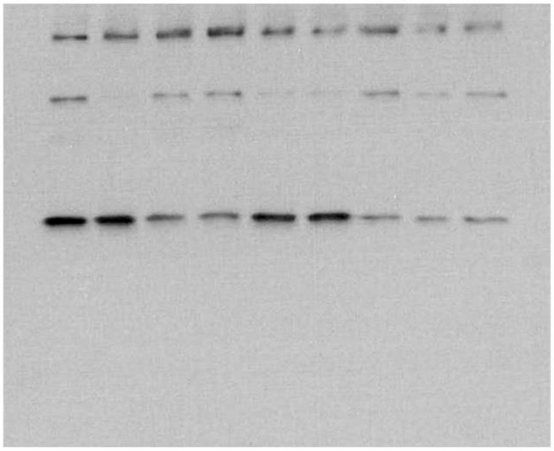

Standard Western Standard Western diet
------ Wild type ------ ----- APP/PS1 ------

β actin

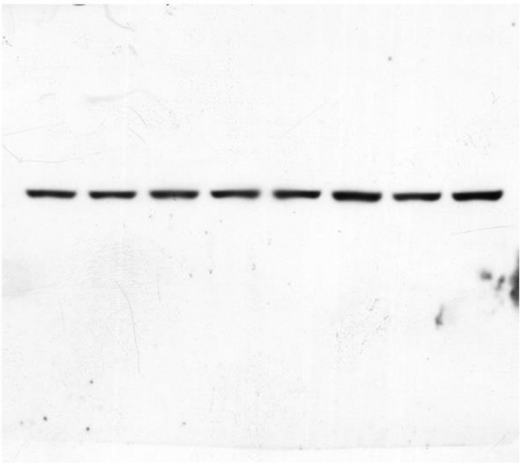

Standard Western Standard Western diet
------ Wild type ------ ----- APP/PS1 ------

Supplementary figure for Figure 1B - Full blots

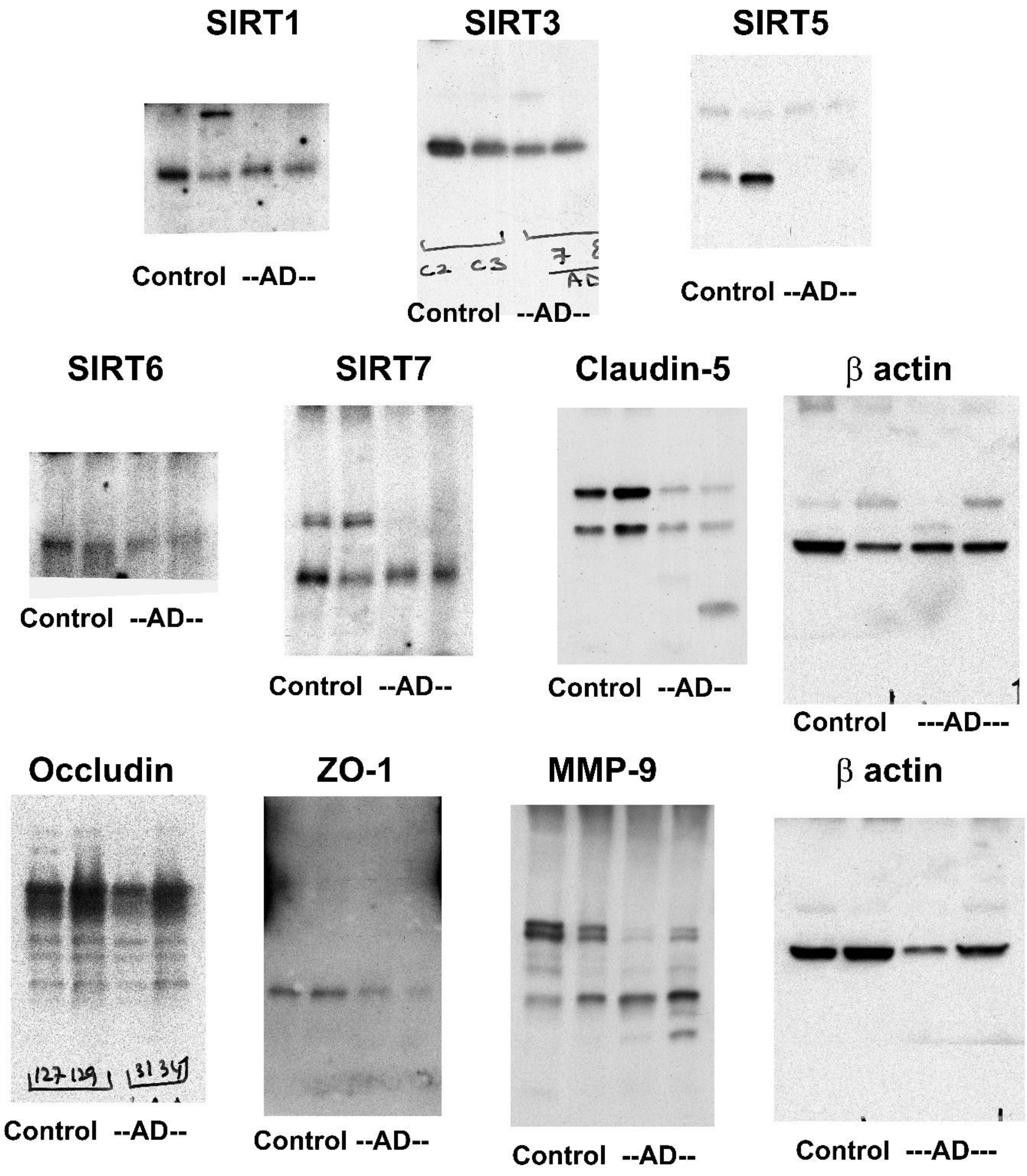

Supplementary figure for Figure 2A - Full blots

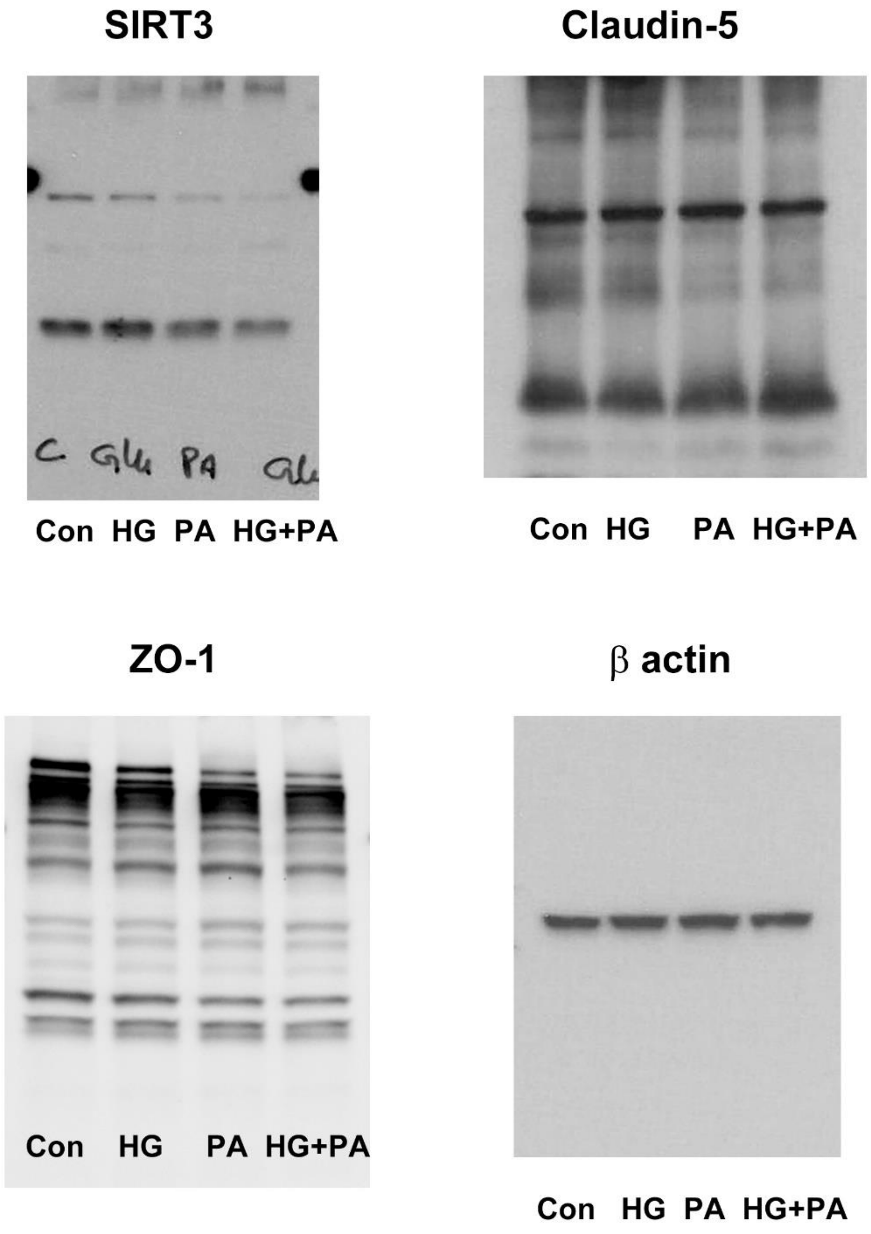

Supplementary figure for Figure 3A - Full blots

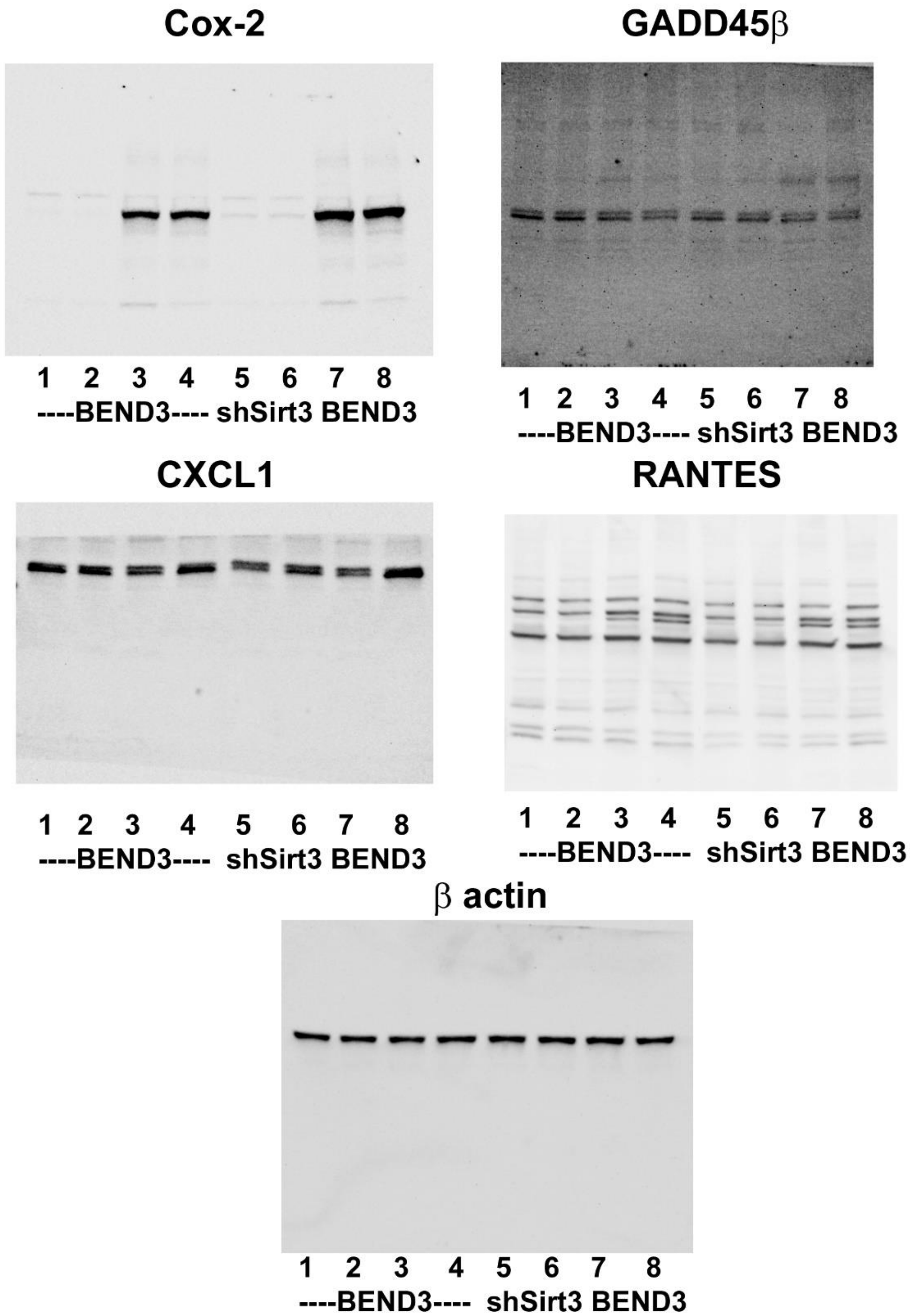

Supplementary figure for Figure 4B - Full blots

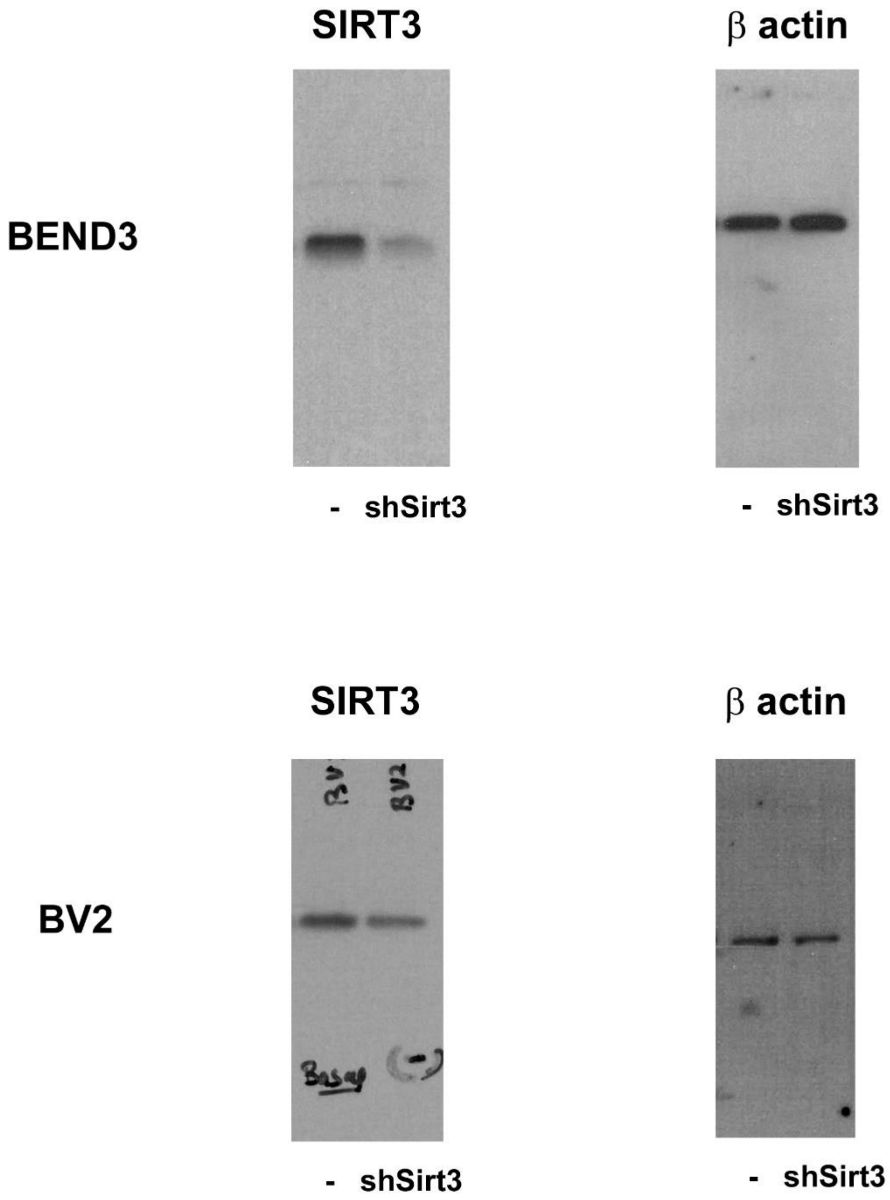

Supplementary figure for Figure 5A - Full blots
